# The Implication of Reticulons (RTNs) in Neurodegenerative Diseases: From Molecular Mechanisms to Potential Diagnostic and Therapeutic Approaches

**DOI:** 10.3390/ijms22094630

**Published:** 2021-04-28

**Authors:** Agnieszka Kulczyńska-Przybik, Piotr Mroczko, Maciej Dulewicz, Barbara Mroczko

**Affiliations:** 1 Department of Neurodegeneration Diagnostics, Medical University of Bialystok, 15-269 Bialystok, Poland; maciej.dulewicz@umb.edu.pl (M.D.); mroczko@umb.edu.pl (B.M.); 2 Department of Criminal Law and Criminology, Faculty of Law, University of Bialystok, 15-213 Bialystok, Poland; p.mroczko@uwb.edu.pl; 3 Department of Biochemical Diagnostics, Medical University of Bialystok, 15-269 Bialystok, Poland

**Keywords:** reticulons, nogo proteins, neurodegenerative diseases, multiple sclerosis, amyotrophic lateral sclerosis, Alzheimer’s disease

## Abstract

Reticulons (RTNs) are crucial regulatory factors in the central nervous system (CNS) as well as immune system and play pleiotropic functions. In CNS, RTNs are transmembrane proteins mediating neuroanatomical plasticity and functional recovery after central nervous system injury or diseases. Moreover, RTNs, particularly RTN4 and RTN3, are involved in neurodegeneration and neuroinflammation processes. The crucial role of RTNs in the development of several neurodegenerative diseases, including Alzheimer’s disease (AD), multiple sclerosis (MS), amyotrophic lateral sclerosis (ALS), or other neurological conditions such as brain injury or spinal cord injury, has attracted scientific interest. Reticulons, particularly RTN-4A (Nogo-A), could provide both an understanding of early pathogenesis of neurodegenerative disorders and be potential therapeutic targets which may offer effective treatment or inhibit disease progression. This review focuses on the molecular mechanisms and functions of RTNs and their potential usefulness in clinical practice as a diagnostic tool or therapeutic strategy.

## 1. Introduction

Reticulon proteins (RTNs) are a family of membrane-bound proteins mediating neuroanatomical plasticity and functional recovery following central nervous system (CNS) injury or diseases [1]. Recent studies have demonstrated that the clinical relevance of RTNs in the development and pathogenesis of central nervous system conditions is the prime focus of scientific interest. However, little is yet known about the biological functions of most of the RTNs. A growing body of evidence indicates that RTNs play a pivotal role in the development and preservation of the CNS as well as synaptic plasticity, although, parallelly, they may act as an inhibitory factor in CNS recovery after damage [2,3]. Moreover, reticulons are involved in multiple cellular processes such as preservation of the endoplasmic reticulum hemostasis, membrane trafficking, apoptosis, calcium homeostasis, and inflammation [4,5]. Therefore, impairment of any of the physiological processes controlled by RTNs may lead to the development of pathological conditions associated with the degeneration of the CNS. It has been revealed that RTNs are involved in the development and progression of some neurodegenerative diseases (ND), such as Alzheimer’s disease (AD), multiple sclerosis (MS), or amyotrophic lateral sclerosis (ALS) or other neurological disturbances, including brain injury or schizophrenia [6,7,8,9,10]. The expression and levels of RTNs change in the course of neurodegenerative disorders, which may indicate their possible diagnostic usefulness. Given that RTNs are crucial factors promoting neurodegeneration and neuroinflammation, it is suspected that suppression of their activity could be a novel therapeutic strategy for these diseases. Understanding the role of RTNs and their interacting partners in the pathogenesis of neurodegenerative disorders is vital since these molecules could potentially have considerable significance in neurodegenerative diseases. The present paper lends insight into the molecular mechanisms and functions of RTNs, particularly RTN-4A (Nogo-A), important in neurodegeneration. Furthermore, it summarises the findings of studies exploring their potential clinical usefulness—as a diagnostic tool or a therapeutic target.

### Characteristics of the RTN Family

The reticulon family is a large and varied group of membrane-associated proteins that consists of over 300 eukaryotic proteins [11,12]. The family is highly conserved as it developed early in eukaryote evolution. The mammalian RTNs are encoded by four independent *rtn* genes (*rtn1-4*) located in different chromosomes [11,13]. Interestingly, in humans, approximately 73% amino-acid identity has been demonstrated between RTN1, RTN3, and RTN4, while RTN2 appears to be less similar to other genes (52% identity with RTN4) [11]. Reticulons have a similar genomic structure—almost all of them contain numerous introns and exons. However, the presence of differential promoters and alternative splicing have led to the production of multiple isoforms both at the mRNA and protein levels (Table 1) [13]. Nogo-A proteins are one of the best known and most extensively investigated proteins among reticulon family members. Moreover, it is believed that the longest transcript of RTN4 proteins, Nogo-A, has the most potent inhibitory effect on the CNS in comparison with other RTNs (RTN1-3 and RTN4-B), which probably arises from the structure of the protein [13,14,15].

All RTNs are composed of a characteristic COOH-terminal reticulon homology domain (RHD, size range 180–200 aa), which consists of hydrophilic 66 amino-acid long loop (called Nogo-66) flanked by two transmembrane 60–70 aa-long hydrophobic regions and the N-terminal domain, which demonstrate significant differences in length and sequence, even within RTNs isoforms [11,14]. The presence of RHD is necessary for interaction with other proteins and, primarily, subcellular localisation [14], while the N-terminal domain is responsible for specific biological functions [15]. Reticulons, particularly Nogo-A, have several functional domains such as Nogo-A-∆20, Nogo-66, Nogo-A-∆2, Nogo-A-ext, Nogo-A-24, Nogo-C39, which allow them to form multi-protein structures with their corresponding receptors (e.g., Nogo receptor 1 (NgR1), Paired immunoglobulin‑like receptor B (PirB)) and to perform various biological functions in other cellular compartments [14].

RTNs are widely present in all tissues of eukaryotic organisms, although each protein of the reticulon family displays a unique expression pattern that corresponds to relevant issue and cell types (Table 1).

Analysis of mRNA has demonstrated that all reticulons (*RTN1-4*) are present in the brain and the expression of the majority of them (*RTN1-A, *RTN1-C*, *RTN2-A*, *RTN2-B*, *Nogo-A*/*RTN4-A)** is highest in nervous system tissues, although members of the RTN1 and RTN4 subfamily appear to be of primary importance for CNS function [14]. Extensive research of the topic has revealed that expression levels of RTN4-A in cells depend primarily on the stage of CNS development. Immunohistochemical studies have shown low RTN4-A/B expression in some types of neurons, particularly those with long axons, which was reduced to almost undetectable levels under physiological conditions in adults [16,17]. High levels of RTN4 have been found in dorsal root ganglia, spinal cord, olfactory bulb, pyramidal cells, and hippocampal neurons [16,18,19,20]. However, the highest expression of RTN4-A has been demonstrated in oligodendrocytes and myelin-forming cells of the adult CNS. Besides the CNS, RTN4-A expression has been found in some peripheral tissues and cells [16] (Table 1). RTN1 (also known as neuroendocrine-specific protein—NSP) is located primarily in neurons of various brain regions and neuroendocrine cells [13,14].

## 2. Role of RTNs in the Nervous System—Molecular Mechanisms

In recent years, significant progress has been made in the understanding of the biochemical and genetic nature of many processes in which reticulons are involved. It is indisputable that the preservation of RTN homeostasis is essential for maintaining normal cell function (Table 2). Reticulons are predominantly intracellular proteins located in the endoplasmic reticulum. RTNs participate in many physiological processes such as shaping the tubular endoplasmic reticulum (ER) network, membrane trafficking, morphogenesis, vesicle formation, as well as cell survival [11]. However, RTNs have also been found on the surface of cells and many types of neurons as crucial factors regulating the development and function of the CNS [4,21].

**Figure 1:**
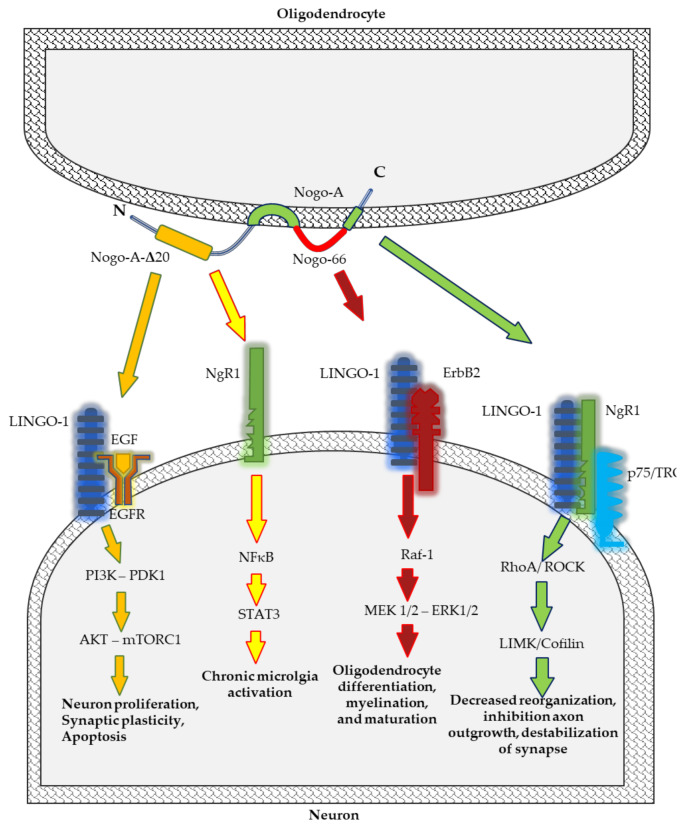
Schematic representation of Nogo-A signalling pathways implicated in neurodegenerative processes.

Among all the RTN family members, RTN4 appears to be the most relevant for physiological and pathological conditions of the CNS. Therefore, we paid particular attention to this protein. Nogo proteins regulate a wide range of CNS cell functions, including development and maintenance of the structure of neurons and oligodendrocytes, axonal growth, myelination, and synaptic plasticity. However, they may also restrict the capacity of axons to grow and regenerate after injury or during disease [21,38] (Figure 1).

Literature data suggest a relationship between intracellular RTNs and apoptosis in neuroprotection and neurodegeneration. RTNs may interact with pro- and anti-apoptotic (B-cell lymphoma 2 protein (Bcl-2), B-cell lymphoma-extra large (Bcl-XL)) proteins by NgR/p75NTR/RhoA signalling pathway [39], simultaneously promoting the survival or inducing programmed death of cells in the brain. Increased levels of RTN3 and RTN4-A in neuronal cells following injury may have a cell-autonomous survival-promoting function [25]. Recent studies on the ischemic, hypoxic, damaged brain of a newborn SD rat model have revealed that increased Nogo-A expression correlates with caspase-3 expression in the hippocamp [40]. Furthermore, inhibition of Nogo-A by using antibodies resulted in inhibition of apoptosis, preservation of the morphological structure and ultrastructure of cells, as well as improvement in neurological function. Nogo-A exerts a protective effect by upregulating Glucose regulatory protein 78 (GRP78), Bcl-2, and C/EBP homologous protein (CHOP), downregulating Bcl-2-associated X protein (Bax) and caspase-12 and finally, suppressing caspase-3 associated with apoptosis [40].

RTNs, especially Nogo-A, are involved in neurogenesis both during the prenatal and postnatal period. The expression of Nogo-A is higher in proliferative cells (such as neuronal and glial cells progenitors) in the early prenatal stages and it decreases to an almost undetectable level in mature astrocytes [41]. During corticogenesis, Nogo proteins regulate tangential neuronal migration, formation and maturation of neurons, as well as remodelling of dendrites through modulating microtubule dynamics [32,41]. In the postnatal period, the expression of Nogo-A changes—it increases in oligodendrocytes and decreases in neurons, except for the neurons from brain areas of high morphological and physiological plasticity, such as the hippocampus, neocortex, subventricular zone/olfactory bulb system [42,43,44].

Nogo-A located on the cell surface regulates neuronal distribution and migration in the early stages of CNS development [41]. In vitro studies have established that Nogo protein affects the distribution of neuronal precursors via modulation of the collapse of the cytoskeleton of neurons and directs them to the target regions of the brain. Nogo-A, by the NgR1–LINGO1 complex, activates the Rho-A/ROCK pathway leading to the rearrangement of a grown cone’s actin cytoskeleton and inhibition of neurite outgrowth, and their repulsion in intact as well as injured adult CNS (Figure 1) [21,31,45].

In hippocampal neurons and the motor cortex, Nogo-A and its receptor (NgR1) negatively modulate synaptic plasticity [36,43]. On the other hand, Nogo-A neutralisation not only enhances synaptic plasticity in the motor cortex but also improves motor learning capacity [2]. However, it is still not fully understood how the Nogo-A blockade influences synapse function. Nogo-A is highly expressed in the adult hippocampus in several prenatal and postnatal neurons, including CA3 and CA1 pyramidal cells [46]. Extensive studies on cell cultures and animal models into the role of Nogo proteins in synaptic plasticity have revealed that suppression of Nogo-A activity results in marked changes in the morphology and volume of dendritic spines of CA3 and CA1 neurons and leads to augmented sprouting of CA3 axons [47]. Morphological changes concern mainly the thickness, length, or complexity of dendrites [48]. Additionally, Nogo-A affects the electrophysiological properties of neurons by modulating the ionic conductance of the cell membrane [2]. Furthermore, Nogo-A appears to act as a stabiliser of synapses—inactivation of this protein leads to increased growth and higher plasticity in axons and dendrites [43]. It has been demonstrated that ablation of Nogo-A or NgR1 by inhibiting antibodies in knock-out animal models increases long term potentiation (LTP) [49]. It is probable that NgR1 regulates the synaptic plasticity mechanism through the RhoA/ROCK pathway as the number of postsynaptic densities (PSDs), which are positive for phosphorylated-cofilin, increases after LTP induction [50]. At the next stage, changes in spine morphology increase LTP and lead to impaired spatial learning ability [51]. Nogo-A may influence cognitive functions, also regulating glutamate receptors in hippocampal neurons [52]. Nogo-A modulates N-methyl-D-aspartate receptor expression (NMDAR) and α-amino-3-hydroxy-5-methyl-4-isoxazolepropionic acid receptor (AMPAR) subunits by the mTOR pathway. Nogo-A decreases the expression of NMDA and AMPA glutamate receptor subunits. Peng et al. have demonstrated that suppression of Nogo-A or NgR1 by siRNA treatment enhances the expression of NMDAR subunits GluN1, GluN2A, and GluN2B, and AMPAR subunits GluA1 and GluA2, as well as the post-synaptic scaffolding protein PSD95 [52]. Notably, chronic inactivation of NMDAR may cause psychosis, cognitive decline, and other manifestations, as in the case of schizophrenia and AD [53]. Moreover, AMPAR trafficking is restricted by Nogo-A-NgR1 signalling, decreasing neural plasticity and subsequently leading to neurodegeneration [54].

Studies on mice deficient in Nogo-A have revealed that this protein is vitally important for differentiation of oligodendrocyte, myelin sheath formation, and axonal growth within the first postnatal month. Considering that Nogo-A regulates oligodendrocyte maturation and the formation of myelin sheaths but does not affect the structure of myelin sheaths and Ranvier nodes, their participation in remyelination processes occurring during pathological conditions, including MS, has been suggested [34]. The molecular mechanism which regulates oligodendrocyte differentiation and myelination depends on leucine rich repeat and Immunoglobin-like domain-containing protein 1 (Lingo-1), a receptor of Nogo-A. In vivo and in vitro studies have demonstrated that Lingo-1 acts as a negative regulator of oligodendrocyte differentiation and myelination [55,56]. Lingo-1 affects receptor tyrosine kinase ErbB2 prevents its translocation into lipid rafts, and inhibits its phosphorylation for activation [57].

Microglia, astroglial cells, and neurons play a crucial role in maintaining homeostasis in the CNS and neuroinflammation. Initially, neuroinflammation is a protective mechanism in CNS cells. However, at a later stage, enhanced inflammatory reaction may lead to degeneration of brain cells. Nogo-A and its receptor NgR, located in microglia, participate in neuroinflammation [58,59]. A neuroinflammatory process in microglia is regulated by the Nogo-66/NgR pathway. The activation of NgR by Nogo induces the expression of proinflammatory cytokines and inhibits cell adhesion and migration [59]. Ullah et al. have demonstrated that overexpression of Nogo-A promotes an increase in pro-inflammatory cytokines such as IL-6 and TNF-α, whereas inhibition of Nogo-A decreases the concentration of these molecules [28]. Nogo peptide, Nogo-P4—the core inhibitory peptide of Nogo-66—stimulates the proinflammatory function of microglia via activation of the nuclear factor kappa-light-chain-enhancer of activated B cells (NF-κB)/ signal transducer and activator of transcription 3 (STAT3) signalling pathway [58]. Moreover, Nogo receptors (NgR1 and NgR2) located on the cell surface participate in the migration of macrophages from inflammation sites in the peripheral nerve [60]. Not only does Nogo-A promote the activation of the inflammatory process mediated by the release of pro-inflammatory cytokines, but it is also a crucial factor regulating the perception of pain associated with inflammation in dorsal root ganglion (DRG) neurons [29,30]. Nogo-A aa 846–861 is a Nogo-A-specific segment and a novel ligand of NgR1 which promotes inflammatory pain [30]. Nogo-A regulates inflammatory heat hyperalgesia by suppressing the transient receptor potential vanilloid subfamily member (TRPV)-1 channel, the endogenous noxious heat transducer during inflammation, and activation of the LIM domain kinase (LIMK)/cofilin signalling pathway. The LIMK/cofilin pathway regulates phosphorylation and actin polymerisation, and polymerised fibres of actins help in the inflammatory response [29].

## 3. Diagnostic Usefulness of RTNs in Neurodegenerative Diseases

Neurodegenerative diseases represent a considerable challenge in terms of clinical diagnosis and treatment. They are still incurable, difficult to detect at early stages because of the asymptomatic incubation period, and hard to differentiate at later stages due to similarities between different neurodegenerative disorders or their atypical forms [61,62]. Neurodegenerative diseases are diagnosed based on established clinical criteria, including the neuropsychological evaluation and neuroimaging methods, such as magnetic resonance imaging (MRI), single-photon emission computed tomography (SPECT), 18F-fluorodeoxyglucose positron emission tomography (glucose-PET) and amyloid-PET, as well as biochemical tests [63]. However, available diagnostic tools are still insufficient [64]. Therefore, biomarkers that would improve the diagnosis of these devastating, heterogeneous diseases are continually sought and urgently needed. Nogo proteins could provide both an improved understanding of early pathogenesis of these disorders and may be potential therapeutic targets, which may offer effective treatment or inhibit disease progression [65]. Potential biomarkers for neurodegenerative disorders should identify presymptomatic patients and monitor disease progression. Currently, there are no commonly accepted biomarkers with proven reliability as a measure of disease burden in neurodegenerative diseases, except for cerebrospinal fluid biomarkers (CSF) biomarkers in Alzheimer’s disease, which is a significant problem in clinical practice [64]. On the other hand, even if a molecular specific biomarker, such as amyloid imaging in AD, is found, it may not fully reflect the pathomechanism of the disease, which may affect its efficacy in pre-clinical disease detection and diagnostic confirmation [66]. Hence, it appears that a single biomarker cannot fulfill all these criteria and therefore current efforts should focus on creating a panel of biomarkers. Other difficulties and hurdles that need to be overcome in investigations on specific and sensitive biomarkers for neurodegenerative diseases are standardised sample processing, methods and protocols, and storage and specialised laboratories [63,67]. Furthermore, a candidate biomarker ought to exhibit clinical utility in prospective studies and be validated in a separate set of samples [61]. To date, a great number of potential biomarkers have been evaluated in the CSF of patients with neurodegenerative disorders. CSF biomarkers are still one of the primary laboratory modalities and should constitute part of a routine diagnostic process of these disorders [67].

**Figure 2:**
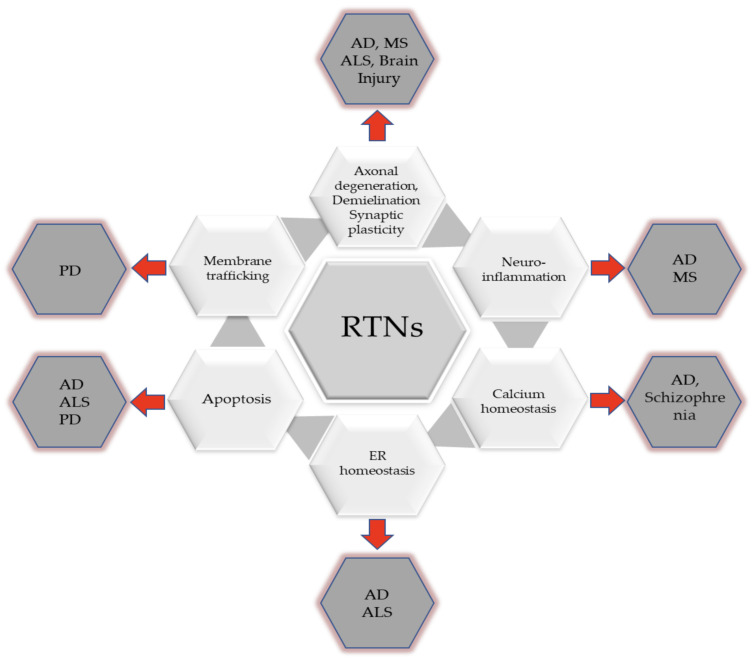
Involvement of RTNs in the development of neurodegenerative diseases.

Neuropathological changes develop gradually over several years before the onset of clinical symptoms. Therefore, it is highly important that they are detected at the earliest possible stage. Many researchers focus on searching for biomarkers reflecting neuropathological changes in the brain that could be applied in clinical practice. Reticulons, neuron-specific proteins released from the brain following axonal degeneration, appear to be candidate biomarkers for neurodegeneration (Table 3). A growing body of evidence indicates that reticulons (RTNs) are involved in neurodegenerative disease [13] (Figure 2). It has been established that RTNs are key factors regulating some processes in the CNS and fluctuations in RTN levels disturb normal brain function in several neurodegenerative disorders, such as AD, MS, ALS, or other neurological diseases (Table 4) [5,68,69]. Moreover, reticulons reflect the extent of axonal damage in the brain and therefore would be helpful in clinical practice as an indicator of overall disease burden, and could potentially predict future disease activity. It appears that RTNs as potential biomarkers provide a dynamic and powerful approach to understanding the spectrum of some of the neurodegenerative diseases with applications in disease diagnosis and prognostication as well as randomised clinical trials. It is postulated that alterations in RTNs levels in the tissues or CSF of patients with ALS or MS may offer a means for improved classification of the disease and its risk factors, and they can enhance our understanding of the biological mechanisms underlying disease pathogenesis. The additional advantage of these proteins is the possibility of reflecting the entire spectrum of the disease, from its earliest manifestations to terminal stages, e.g., in ALS.

### 3.1. RTNs in Multiple Sclerosis

A combination of the heterogeneous and complex pathogenesis of MS with an uncertain prognosis makes identification of disease-specific diagnostic and prognostic biomarkers necessary. Furthermore, limited availability and lack of sensitivity of commonly used diagnostic tools, including MRI and oligoclonal immunoglobulin G bands, make them insufficient for the diagnosis of the disease [74]. Therefore, it is crucial that relevant biomarkers are found. The main hallmarks of the disease are multifocal inflammatory demyelination and axonal damage in the CNS white matter. Studies have suggested that Nogo-A and its receptor NgR could be potential biomarkers for MS [68,70,71,72,75,76]. Increased levels of Nogo-A have been found in the brain tissues, cerebrospinal fluid, and blood of patients with MS MS [68,70,71,75].

Early studies in experimental autoimmune encephalomyelitis (EAE), a widely used animal model for MS, demonstrated a high expression of Nogo-A in the brain [77,78]. An immunohistochemical study by Satoh et al. confirmed Nogo-A overexpression in oligodendrocytes at the edge of chronic active demyelinating lesions in patients with MS, and unexpectedly marked immunoreactivity for NgR in reactive astrocytes and microglia/macrophages in these lesions in comparison with their expression in the white matter of brains of neurologically normal subjects. The above findings indicate that both the ligand and receptor of the Nogo-A protein may play an essential role in the pathogenesis of the disease [70]. In agreement with this study, other studies using Western-blot analysis have demonstrated the presence of Nogo-A in autopsied brain tissue and CSF of patients with MS. Interestingly, the expression of a soluble Nogo-A fragment was found exclusively in patients with MS (in over 90% of CSF samples from patients with MS) in comparison to meningo-encephalomyelitis, other neurological diseases, and autoimmune disorders [68]. Moreover, the authors reported that Nogo-A was present in both types of the disease: remitting-relapsing MS (RR-MS) as well as secondary progressive MS (SP-MS). Interestingly, CSF expression of Nogo-A was revealed in the whole spectrum of the disease (at the onset of MS, in long-lasting and advanced cases). However, no relationship between CSF Nogo-A and disability assessed using the Expanded Disability Status Scale (EDSS) scale was established. The authors postulated that a soluble fragment of Nogo-A could be a relevant and specific biomarker for multiple sclerosis [68]. However, inconsistent results have been reported in the available literature [79].

Nogo receptor-1 ligand lateral olfactory tract usher substance (LOTUS) has also attracted attention both as a marker of disease activity and a hopeful therapeutic target for multiple sclerosis [72]. It has been established that LOTUS is an endogenous NgR1 antagonist which inhibits Nogo activity, preventing NgR1 binding with necessary ligands such as myelin-associated glycoprotein, oligodendrocyte myelin glycoprotein, B lymphocyte stimulator, and chondroitin sulfate proteoglycans. Thus, it prevents the formation of complexes and suppresses the Nogo-A signalling pathway, which consequently promotes axonal regeneration and functional recovery [80,81]. On the other hand, a decrease in LOTUS stimulates NgR1 signalling and triggers the pathological cascade, leading to the degeneration of neurons [72]. In patients with MS, the diagnostic and prognostic value of LOTUS levels in the CSF has been confirmed. Takahashi et al. revealed significantly decreased CSF LOTUS levels in patients with relapsing-remitting MS (RRMS) and secondary progressive MS (SPMS) in comparison to subjects with ALS, multiple system atrophy, and normal controls. Furthermore, the concentration of this protein was correlated with disease activity. In the remission group of patients with RRMS, the LOTUS level was increased and was similar to that of healthy controls, in contrast to the relapse group of patients with RRMS [72]. These findings confirm that decreased CSF LOTUS levels could be a useful marker of disease activity and prognosis [72,74]. Additionally, a negative correlation between mRNA and protein levels of LOTUS, and disease activity as well as the extent of neuroinflammation, indicate the usefulness of this marker for accurate diagnosis and prognosis in both early and progressive phases of MS [72,74]. The authors claim that fluctuations in LOTUS levels might reflect the degree of axonal degeneration and the extent of neuroinflammation, which are responsible for the progression and functional disability in MS [74,82]. These findings confirm that CSF LOTUS levels might reflect disease burden and provide a better understanding of the pathogenesis of MS.

Biomarkers that can be measured in peripheral blood are of particular clinical importance since obtaining blood samples is an easy and non-invasive procedure. However, blood biomarkers have some limitations in the diagnosis and prognosis of neurodegenerative diseases (e.g., they display lower sensitivity) compared to CSF biomarkers, which reflect the pathology of the disease restricted to the CNS more accurately. Despite these limitations, peripheral blood biomarkers can provide relevant information on immune factors that cause MS and the therapeutic efficacy of drugs [83]. In the face of this knowledge, it seems essential to quote a recent Swedish study that has indicated the possibility of using plasma RTN3 as a potential predictive biomarker for assessing treatment outcomes in MS. It was reported that plasma levels of nine out of 59 tested proteins decreased during treatment with natalizumab, with RTN3 and PEBP1 displaying the most significant changes [71]. Moreover, it was only RTN3 levels that differed between healthy controls and patients with MS before and during treatment. Nevertheless, the authors were not able to confirm the obtained results using a different method [71]. Therefore, further studies are necessary to explore this issue and detect potential biomarkers which could be used to facilitate the treatment decision-making process.

### 3.2. RTNs in Amyotrophic Lateral Sclerosis

Amyotrophic lateral sclerosis (ALS) is a rapidly progressing, fatal neurodegenerative disease. The main feature of the disease is the loss of both upper and lower motor neurons in the brain and CNS, including the brain stem and the spinal cord, generally leading to gradual weakness and atrophy of skeletal muscles [84]. Since available diagnostic tools are insufficient, novel biomarkers are sought. Discovering biological markers enabling early detection of the disease is highly important as it would allow for assessment of patient prognosis and early initiation of neuroprotective treatment.

Mounting evidence suggests that reticulons, particularly members of the RTN4 (Nogo) subfamily, are involved in the pathogenesis of ALS [73,85,86]. Studies in mouse models and patients with ALS have revealed that Nogo proteins are ectopically expressed on affected skeletal muscle and contribute to disease development [69,73,85]. Jokic et al. has demonstrated a relationship between early overexpression of RTN4A in muscle fibres of ALS mouse models and the impairment of the neuromuscular junction and subsequent motor neuron axon degeneration [73]. The authors reported that Nogo-A overexpression in muscle fibres destabilises neuromuscular junctions, causing terminal retraction and denervation with axon and motor neuron death, and muscle atrophy. On the other hand, they found that deletion of Nogo-A reduces muscle denervation and prolongs the survival of mice with ALS [73]. Other authors have confirmed that the receptor for Nogo-A (NgR) could also be a promising clinical target [87]. It has been demonstrated that binding of the Nogo-66 receptor (NgR) by both agonistic (Pep4—amino acid 31−55 of Nogo-A) as well as antagonistic Nogo-66-derived peptide (NEP1—amino acid 1−40 of Nogo-A) could preclude apoptotic signalling and protect against p75^NTR^ dependent motor neuron death [88]. These findings indicate that RTN4 could reflect the burden of neurodegeneration and, importantly, may be a promising therapeutic target.

According to some authors, Nogo-A expression may be a useful biomarker for identifying ALS in the early stages, when the diagnosis is difficult to confirm [69,86]. It was revealed that mRNA expression of three isoforms of RTN4 is altered in both postmortem muscle tissue samples of patients with ALS and in ALS mouse models expressing human superoxide dismutase (SOD) with a disease-causing dominant mutation in comparison with wild-type mice [86]. Increased mRNA levels of RTN4A and, to a lesser degree, RTN4B, were observed in pre-symptomatic animals, while RTN4C was overexpressed exclusively in asymptomatic mice [88]. Interestingly, RTN4A and RTN4B levels, barely detectable in healthy adult muscles, increased in ALS mice and correlated with disease severity [73], whereas overexpression of RTN4C isoform decreased [86]. In agreement with these findings are other studies which have provided evidence that muscle Nogo-A expression could be a prognostic marker in the lower motor neuron syndrome (LMNS) [69]. LMNS is considered the initial stage of ALS. Enhanced Nogo-A expression in biopsy samples from patients with LMNS allowed for identification of patients at higher risk of progressing to ALS. Moreover, altered muscle Nogo expression can be detected three months after the onset of symptoms. Nogo-A may not only be an early indicator of the disease but also an adverse prognostic factor in patients with ALS. ALS-related denervation was excluded in Nogo-A-negative patients. However, Nogo-A-negative patients with progressive spinal muscular atrophy (PSMA) and slow degenerative LMNS had a slower progressive course of the disease with mild functional impairment [69]. Importantly, Nogo-A examination was a laboratory test characterised by high diagnostic power. Inability to detect the disease early results in delayed initiation of therapy and worse outcomes. Hence, it is essential that such tests are implemented in clinical practice to reduce the waiting time for treatment or inclusion in clinical studies [69]. However, the disadvantage of the above study was the invasiveness of muscle biopsy. Therefore, another group of researchers assessed the level of this protein in the serum of patients with ALS [89]. Since in physiological conditions, Nogo-A is a transmembrane protein that is not normally secreted, the authors postulated that atrophic changes related to ALS may lead to the release of Nogo-A into the blood. Unexpectedly, serum-based findings revealed a trend toward lower Nogo-A concentrations in patients with ALS in comparison with healthy controls [89]. The authors indicated that serum Nogo-A levels may increase in the early phase of the disease and decrease in the advanced stage, the stage at which blood samples were collected from patients with ALS [89]. Further studies are required to explain this phenomenon.

In the light of the above-mentioned findings, Nogo-A and its receptor NgR are upregulated in muscles and spinal cord motoneurons and play an essential, neuropathological role in ALS. Furthermore, studies have confirmed that Nogo-A could be used as an early diagnostic and prognostic marker for ALS, although the disadvantage of the biomarker is that it is not specific to this disorder. On the other hand, the possibility of using Nogo and NgR as potential therapeutic agents for patients with ALS is being considered [90].

### 3.3. RTNs in Alzheimer’s Disease

Alzheimer’s disease (AD) is a highly heterogeneous and complex disease. Despite the fact that substantial progress has been made in research on the biology of AD, the precise mechanism of the disease is not well understood. At present, many hypotheses explaining the pathogenesis of AD exist. Some evidence suggests that all RTN proteins (RTN1-RTN4) and receptor NgR are engaged in the pathology of AD by regulating the beta-site amyloid precursor protein-cleaving enzyme 1 (BACE1) function or APP processing, and thereby amyloid β production in the brain [17]. To date, the greatest research interest has been focused on RTN3 and RTN4 proteins [91] as well as the NgR receptor [6,92] which affects Aβ production and deposition.

RTN3 appears to be a potential marker for AD since it has been demonstrated to oligomerise and gather in a subpopulation of dystrophic neuritis in postmortem AD brain tissue [93,94]. In vitro studies have demonstrated the presence of dystrophic neurites in transgenic mice overexpressing RTN3. Furthermore, these neurites were morphologically similar to those observed in AD brains and, interestingly, were associated with the development of RTN3 aggregates in susceptible brain regions [93,94]. On the other hand, contradictory findings have been presented by Kume et al. who, despite reporting that RTN3 co-locates subcellularly in the same space as BACE1, did not detect significant differences in its expression levels between control and AD brains [95]. The relevance of RTNs for the pathogenesis of AD and their exact role is not fully understood and remains to be established. Based on available findings, we know that RTN3 and RTN4 interact with BACE1 (β-secretase) and inhibit its activity, which results in decreased production of amyloid β [91,92], while NgR interacts with amyloid precursor protein (APP) to reduce the production of peptide Aβ [6].

The role of RTN3 in the regulation of BACE1 activity and production of amyloid deposits has been confirmed in in vitro and in vivo studies [92]. RTN3 overexpression has a neuronal protective effect, is associated with decreased APP processing at the β-secretase site, and leads to a reduction in BACE activity while RTN3 suppression favours amyloid deposition [88,92]. Moreover, the overexpression of RTN3 may reduce axonal transport of BACE1, decreasing the level of this secretase in axonal terminals and, in consequence, leads to a reduction in amyloid deposition [96,97]. Kume et al. investigated molecular mechanisms by which RTNs regulate BACE1. They reported that it is solely the two-transmembrane-domain tertiary structure of RTN proteins that is critical for the ability of RTNs to modulate BACE1 activity [95]. Biochemical and animal model studies have revealed that RTN3 deficiency increases BACE1 levels, and thereby enhances the synthesis of toxic peptides of Aβ_42_ [94]. However, it appears that the effect of RTN3 deficiency on amyloid-beta production and generation is not so significant, probably due to compensation by other RTN members in neurons [94]. In studies on mouse models, Shi et al. demonstrated that RTN1 and RTN3 strictly regulated as RTN1 deficiency causes an increase in RTN3 levels. Furthermore, in RTN3-null mice, higher levels of BACE1 protein have been observed while RTN1 deficiency has been found to exhibit a far less marked effect on BACE1 expression and its association with dysmorphic neurites in Alzheimer’s amyloid plaques is not so strong. The authors postulated that a more prominent role of RTN3 in the pathology of AD is related to unique sequences of this protein and its different localisation in neurons [94]. In agreement with these findings are genetic studies which, by using mutation screenings of the RTN3 gene, explored whether RTN3 variants may influence the pathogenesis of AD. In patients with sporadic early-onset AD and sporadic late-onset AD, multiple RTN-3 variants, mainly in the 5′ non-coding region and the N-terminal domain of RTN-3, were found. Two variants, RTN3 c.-8G > T and c.116 C > T, appear to be the most important. The RTN3 c.-8G > T variant seems to be responsible for reduced expression of RTN3, while the second variant, c.116 C > T, by causing a change in amino acid sequence T39 M, impairs axonal transport of BACE1 in cultured neurons and may be involved in Aβ secretion at synaptic sites [98].

Interestingly, overexpression of RTN4B and RTN4C, similarly to RTN3, reduces the secretion of Aβ40 and Aβ42 by approximately 30–50% [91]. By contrast, immunoreactivity of RTN4A and its receptor NgR (Nogo-66 receptor) facilitates Aβ secretion in the brain [6]. It has been demonstrated that overexpression of RTN4A and Nogo-66 receptor in the hippocampal neurons of patients with AD is related to the formation of both senile plaques [6] and neurofibrillary tangles [99]. Xiao et al. revealed that enhanced expression of RTN4A and activation of Nogo-P4, the Nogo-66 active fragment, inhibits neurite outgrowth and promotes Aβ peptide generation and excretion simultaneously, which, in turn, might lead to the onset and development of AD [6]. Moreover, Nogo-P4 may act as a positive modulator of Aβ production by activating downstream signalling molecules, ROCK and PKC. The authors reported that activation of the ROCK pathway increases Aβ42, whereas PKC activation decreases the Aβ_42_ level. Activation of ROCK seems to inhibit the non-amyloidogenic processing of sAPPα, which probably triggers the amyloidogenic metabolism of APP and increases production of Aβ_42_ [6].

Moreover, RTN3, RTN4A, and RTN4B proteins may influence the pathogenesis of AD, acting as negative regulators of synaptic plasticity and factors modulating development of the impairment of cognitive functions [36,49]. A recent study using an AD mouse model has revealed that RTN4B overexpression in hippocampal neurons ameliorates abnormal Aβ accumulation and cognitive impairment. BACE1 activity is regulated by sirtuin 2 protein (SIRT2). SIRT2 causes RTN4B deacetylation, which results in ubiquitination and degradation of RTN4B, and then damaged RTN4B interacts with BACE1 and reduces the production and aggregation of Aβ. Furthermore, the authors have demonstrated that inhibition of SIRT2 or overexpression of RTN4B ultimately alleviates cognitive decline in AD [100]. RTN3 negatively impacts on synaptic activity by induction of caspases 8 and 3 [101]. RTN3 and RTN4A regulate basic mechanisms responsible for learning and remembering new information and processes of long-term potentiation (LTP) and long-term depression (LTD). It has been reported that administration of soluble Nogo-66 suppresses long-term potentiation (LTP) and improves long-term depression (LTD). In contrast, neutralisation of endogenous Nogo-A or NgR1, or knockdown Nogo-A increases LTP and improves memory processes simultaneously [102]. In agreement with the above-mentioned findings are studies by Xiao et al. conducted on APP transgenic mice, which have demonstrated that deletion of Nogo-A improves learning and memory deficits, simultaneously reducing AD-related pathology [6].

Given the pivotal role of RTN3 and RTN4 in the modulation of BACE1 activity and generation of amyloid β in AD, inhibition of RTN3 aggregation appears to be a promising therapeutic strategy in the treatment of AD.

## 4. Nogo and Their Interacting Partners as Potential Therapeutic Targets

An improved understanding of the physiological and pathological role of RTNs would allow for the design of rational therapeutic approaches to target neurodegenerative diseases [5]. Pre-clinical and clinical evidence indicates that Nogo-A or NgR1 inhibitors could be novel drug candidates for some neurodegenerative diseases [90,103,104]. Three different antibodies (GSK1223249, NG-101, AXER-204) which effectively restore the function of damaged nerve fibres have been developed. Several clinical studies have been conducted over the past decade to evaluate the usefulness of Nogo-A in clinical practice as a potential biomarker or therapeutic target in patients with MS, ALS, traumatic brain injury, and spinal cord injury. The results from clinical studies in patients with spinal cord injury and ALS demonstrated that treatment with anti-Nogo-A antibodies (administered intravenously or intrathecally) is safe and well-tolerated by patients [90,105].

Robust pre-clinical data indicate that vaccination with anti-Nogo-A antibodies or passive immunisation against Nogo-A reduces clinical symptoms, demyelination, and axonal damage in MS [105]. Therefore, from a clinical viewpoint, it seems crucial that anti-Nogo-A antibodies are used, which may be instrumental in developing a potential novel anti-MS therapy. Currently, immunomodulatory drugs are mainly used to treat relapsing-remitting (RRMS) and primary progressive (PPMS) forms of MS. However, there is a lack of therapeutic approaches for secondary progressive MS (SPMS), a stage of the disease that follows RRMS. Moreover, available therapy options, which suppress or ablate the patient’s immune system, only slow disease progression but do not reverse neurological deficits [106]. Therefore, to improve treatment effectiveness and enhance patients’ quality of life, there is a pressing need for novel therapeutic approaches to restore neuronal function via promoting regeneration and remyelination. One particular regenerative candidate appears to be NgR(310)ecto-Fc fusion protein. Mounting evidence indicates the potential therapeutic utility of a soluble fragment of NgR1 (NgR(310)ecto-Fc) in neurological recovery following CNS disease or injury [107,108]. This fusion protein contains the ligand-binding domain that attracts and blocks all three myelin inhibitors, Nogo-66, MAG, OMpg, from interacting with their cognate receptor, NgR1, and it also prevents the activation of the RhoA-GTP/ROCKII cascade. NgR(310)ecto-Fc antagonising NgR1-dependent signalling in neurons prevents phosphorylation of the downstream microtubule-associated protein and promotes neuronal microtubule and neurofilament stability [109,110]. In vitro studies using animal models have demonstrated that the fusion protein, through the combination of the C-terminal peptide with the Fc fragment of immunoglobulin, facilitates the attachment of additional Fcy receptors to immunological cells, resulting in the enhancement of phagocytosis near or within areas of demyelinating lesions. Early studies revealed that human immunoglobulins in mouse sciatic nerves had a better ability to remove lesional debris, in particular myelin-associated inhibitory factors (MAIFs) [111]. Moreover, a local supply of NgR(310)-Fc to injury sites enhances CNS repair via promoting sprouting of uncut fibres outside injury areas and increasing axonal regeneration following the blocking of MAIFs activity [112]. In agreement with the above findings are the results obtained by Wang et al. who demonstrated that the continuous intracerebroventricular infusion of the human NgR(310)-Fc into rats following spinal cord injury resulted in locomotor recovery [113].

In vivo and in vitro studies have confirmed that treatment with anti-Nogo-A antibodies is generally well tolerated [90]. GlaxoSmithKline (GSK) has developed a humanised anti-NogoA antibody (GSK1223249, Fc-disabled, an anti-NogoA monoclonal antibody against N-terminus) called Ozanezumab. Comprehensive pre-clinical toxicological studies into intrathecal Ozanezumab administration were performed in rodents, non-human primates, and female rabbits. The studies did not find any cardiovascular, respiratory, neurobehavioural, immunogenic, reproductive, or delayed toxicity effects in tested species following treatment with Ozanezumab [90,114]. A few clinical trials with Ozanezumab have been conducted in populations of patients with MS and ALS. Phase II trials evaluate the effect of Ozanezumab on the physical function and survival of individuals with ALS over a treatment period of 48 weeks. Functions were measured using the Amyotrophic Lateral Sclerosis Functional Rating Scale—Revised (ALSFRS-R). Furthermore, patient quality of life, and the safety, tolerability, immunogenicity, and pharmacokinetics of Ozanezumab were also assessed. A completely novel therapeutic strategy for the treatment of neurodegenerative disorders, including ALS, is inhibition of Rho Kinase (ROCK) with Fasudil. Previous studies using cellular and animal models of ALS and other neurodegenerative diseases have revealed that Fasudil exerts a neuroprotective effect, induces axonal regeneration, and improves survival and behavioural outcomes [115].

Antibodies against Nogo-A also seem to be a promising therapeutical strategy that may improve recovery following spinal cord injury (SCI) [104,116]. Phase I and II clinical studies evaluating the safety, tolerability, pharmacokinetics, and efficacy of anti-Nogo-A antibodies (NG-101, AXER-204) in patients with spinal cord injury are currently underway [117]. Encouraging results from a previous trial performed in 52 subjects from two European sites (Germany and Switzerland) pave the way for further studies. Current clinical studies test the efficacy and adverse effects of treatment with anti-Nogo antibodies administered intrathecally. Patients with an acute spinal cord injury (up to 28 days following the event) with complete (no function below the damaged spinal cord) or incomplete (some function remaining below the injury site) functional impairment were enrolled in the study. The primary goal of the novel treatment is to restore the function of damaged nerve fibres, and improve motor and sensory function and patient quality of life. Kucher et al. reported that treatment with anti-Nogo-A antibodies improved motor skills in four out of 19 tetraplegic patients in week 48. Moreover, conversion of almost half of the study group from complete to incomplete SCI was observed [116].

Antibody against the Nogo-66 receptor component, LINGO (Opicinumab—human monoclonal antibody against LINGO-1) has also been assessed in clinical studies with MS patients. However, preliminary results did not fully confirm previous findings obtained in animal model studies [118]. It could be related to a number of factors, e.g., complicated trial design, too heterogeneous a group of patients, or inappropriate tests evaluating treatment effects. A combination of immunomodulatory, neuro and myeloprotective, and neuroregenerative (i.e., neutralising Nogo-A) treatments could yield improved outcomes for patients with MS, particularly in the progressive phase of the disease. Clinical studies testing new therapies for CNS diseases must take into consideration many additional factors. A major challenge in testing drugs for CNS disorders arises from the need to overcome the BBB barrier in order to access CNS tissue, which can be achieved by intrathecal or cerebrospinal fluid administration. It seems that intrathecal administration of drugs, on the one hand, may be associated with achieving maximum doses in the CNS and, on the other, with minimal systemic exposure to the drug, which in turn may reduce the risk of systemic side effects. Moreover, one of the most critical issues is proper study design, including the selection of an appropriate group of patients and methods of assessing treatment effectiveness. Biomarkers reflecting different mechanisms of the development of neurodegenerative diseases could be a necessary tool for diagnosing the disease, forecasting its activity and evaluating the effectiveness of a tested therapy. Hence, a search for biomarkers reflecting CNS degeneration and repair mechanisms which could improve disease detection and facilitate the evaluation of treatment effectiveness appears to be of prime importance.

CSF constitutes the most valuable source of potential biomarkers in neurodegenerative diseases since it reflects neuropathological structural changes in the brain and the biochemical changes that may be associated with them. Determination of the concentration of potential novel biomarkers in CSF requires a lumbar puncture, a procedure which has certain medical and legal limitations. Since it is more invasive than obtaining a blood sample, it requires additional consent from the patient, which is regulated differently in different countries [119]. The norms of medical law, intel alia, define the relationship between medical professionals and their patients, and regulate the performance of clinical procedures, such as the collection of cerebrospinal fluid by lumbar puncture. A lumbar puncture is a medical procedure that can be invasive to varying degrees and may involve a potential risk for the patient. Performance of clinical procedures is regulated not only by medical law but also by criminal law. This is particularly important in the case of individuals with neurodegenerative diseases who are not always able to consent to additional medical procedures concerning them, including a lumbar puncture. Therefore, the role of reticulons in the pathogenesis of neurodegenerative diseases and the possibility of their determination in CSF and blood addressed in this paper involves both medical and legal issues.

## 5. Conclusions and Future Directions

Despite the fact that all RTN proteins are expressed in the brain, RTN4A and RTN3 are assigned the most significant role in the physiological and pathological processes in the CNS. The overexpression of RTN4A is related to many critical neuropathological processes leading to the development of neurodegeneration in the CNS. A deeper understanding of the molecular mechanisms regulated by RTNs, particularly RTN4A, could be crucial to enhancing the knowledge of neuropathological processes underlying some neurodegenerative diseases. Given that changes in the levels of Nogo-A and its corresponding receptors, such as Lotus-1, may reflect the development and even progression of MS or ALS, their potential usefulness as new biomarkers for early diagnosis and prognosis of these disorders is indicated. However, there is still insufficient evidence to support the utility of RTN4A as a biomarker that could improve the diagnostic process of neurodegenerative diseases. Furthermore, the limitation of this biomarker lies in the fact that it is not specific to one neurological disorder. The present paper provides insight into some mechanisms of action of RTNs in physiological and neuropathological processes and summarises the results of studies regarding the possibility of clinical application of Nogo-A proteins. Experiments on cell lines and animal models have revealed that inhibition of the expression of RTN4A or its receptor NgR1 could result in neuroprotective effects and may even restore the function of damaged neurons. Clinical studies evaluating the effectiveness of anti-Nogo-A in treating patients with ALS or spinal cord injury hold the greatest promise. However, clinical studies with anti-Nogo-A antibodies in patients with MS have not fully confirmed previous findings obtained in animal models. The beneficial effect of anti-Nogo-A antibodies in some neurological conditions could be a breakthrough in the search for new therapeutic strategies.

Despite the fact that RTNs, particularly RTN4A, have been extensively studied, there is still insufficient knowledge regarding the dynamics of changes in the concentrations of these proteins in the CSF and blood in the course of various neurodegenerative diseases. It is important that their usefulness as candidate biomarkers is verified in order to increase diagnostic accuracy, allowing for earlier diagnosis, better classification of patients, as well as monitoring of disease activity and treatment. There is also limited data in the available literature on factors influencing the concentration of these proteins in biological materials, which may also be crucial in research into their clinical application.

## Figures and Tables

**Table 1:** Characteristics of the RTN family members.

Family of RTN Proteins	Size (Number of Amino-Acids, aa)	Gene	Transcript Variants	Localisation	
RTN1	208–780 aa	14q23.1	RTN1-A/Nsp-A RTN1-B/Nsp-B, RTN1-C/Nsp-C	Brain, spinal cord, retina	Neuroendocrine tissues, blood cells, spleen
RTN2	205–545 aa	11q13.32	Three main isoforms: RTN2-A/Nspl1-A, RTN2-B/Nspl1-B, RTN2-C/Nspl1-C, and three additional minor isoforms.	Brain, spinal cord, muscle, placenta	Lung, heart, adipose tissue
RTN3	167–945 aa	11q13	Eight gene products available, although only five are present at protein levels RTN3-A/RTN3-A4b, RTN3-A1, RTN3-A2, RTN3-B, RTN3-C	Brain, olfactory nerve, retina, colon, pancreas, thymus	Ovary, placenta, skin, cervix, mammary gland, blood cells
RTN-4/Nogo	199–1192 aa	2p16.3	Three major transcripts: RTN4-A/Nogo-A, RTN4-B/Nogo-B, RTN4-C/Nogo-C and probably above seven additional minor isoforms.	Brain, spinal cord, retina, adrenal gland, colon, skin, skeletal muscle, heart, testes, macrophages	

**Table 2:** Functions of RTNs in the nervous system.

RTN Protein	Functions	Effect	Model	Ref.
RTN1-C/ Nsp-C	Regulate apoptosis	Inhibit protein BCL-X_L_ and favour apoptosis.	Human neuroblastoma cells	[22]
RTN3 RTN4-B/ Nogo-B		Protect against cell death by enhancing anti-apoptotic activity Bcl-2.	Normal HeLa cells SH-SY5Y neuroblastoma cells	[23,24,25]
RTN4-A/ Nogo-A and RTN4-B/ Nogo-B	Form and maintain ER tubules	Shaping and maintenance of normal, tubular morphology of the ER. Overexpression leads to deformation of cell shape, while depletion of Nogo-B results in the formation of peripheral ER sheets.	Cultures of epithelial, fibroblast, neuronal cells	[26]
RTN4-B/ Nogo-B	Macrophage-mediated inflammation	Nogo-B is involved in macrophage infiltration and tissue repair. Endogenous Nogo coordinates macrophage-mediated inflammation with arteriogenesis, wound healing, and blood flow control.	Nogo-knockout mice	[27]
RTN4-A/ Nogo-A	Induce inflammation	Overexpression of Nogo-A leads to upregulation of CHOP, pro-inflammatory cytokines such as IL-6 and TNF-α, while inhibition of Nogo-A results in downregulation of these molecules in myofibres and macrophages.	Murine myoblast cell line (C2C12), Macrophages from bone marrow	[28]
RTN4-A/ Nogo-A	Promote pain perception, neuroinflammation	Nogo-A enhances inflammatory heat hyperalgesia by maintaining TRPV-1 function via activation of the LIMK/cofilin pathway in the rat dorsal root ganglion neuron.	Adult rats with hind paw inflammation D.R.G. culture, primary culture of cortical neurons	[29,30]
RTN4-A/ Nogo-A	Regulate development and distribution of cortical neurons	Regulate radial migration of neuronal precursors and influence motility of these cells during cortical development. Neutralisation of Nogo-A via antibodies or KO Nogo-A increased neuronal precursors motility.	Embryonic mouse cortex	[31]
RTN4-A/ Nogo-A	Regulate the development of neurons (fasciculation, branching and extension)	RTN4A is a neurite growth regulatory factor during the development of CNS, negatively regulates axon-axon adhesion and growth, and facilitates neurite branching. Modulate neurite fasciculation, branching, and extension in the developing nervous system—neutralisation of neuronal Nogo-A, NgR, and LINGO1, or Nogo-A gene ablation leads to longer neurites, increased fasciculation, and decreased branching of cultured dorsal root ganglion neurons.	Cultured dorsal root ganglion neurons from newborn wild-type and Nogo-A KO mice	[21]
RTN4/ Nogo-A	Modulate axon growth	Involved in axonal growth and dendritic modelling through the regulation of microtubule dynamics.	Olfactory epithelium explants from rat embryos	[32]
RTN4/ Nogo-A	Inhibit axonal outgrowth and regeneration in CNS	Suppression of axonal outgrowth and regeneration in CNS via NgR. Neutralisation of Nogo-A or NgR or Rho-A/ROCK pathway enhances regenerative and compensatory fibre growth and improves functional recovery after CNS injury.	Adult rat	[33]
RTN4/ Nogo-A	Regulate oligodendrocyte differentiation, maturation and myelination	Regulate oligodendrocyte differentiation, myelin sheath formation, and axonal growth in physiological conditions. Deletion of Nogo-A significantly delays all these processes in newborn mice. Moreover, RTN4 may influence remyelination in pathological conditions.	Optic nerves and cerebella from mice deficient in Nogo-A (Nogo-A(-/-)	[34]
RTN4/ Nogo-A		Antibodies blocking Nogo-A or NgR inhibit differentiation and maturation of oligodendrocyte progenitors.	Oligodendrocyte progenitor cells (OPCs) and Oligodendrocytes	[35]
RTN4/ Nogo-A	Modulate synaptic plasticity and cognitive function	Suppression of Nogo-A or NgR by miRNA or blocking antibodies increases LTP and enhances synaptic capacity in the hippocampus and motor cortex. Treatment with anti-Nogo-A antibodies improves motor skill learning in adult rodents.	Transgenic rat model with RNAi Adult rodents treated with blocking anti-Nogo-A Abs	[2,36]
RTN4/ Nogo-A	Suppress angiogenesis in CNS	Negative regulator of angiogenesis in the developmental stage of CNS. Domain Nogo-A-Δ20 inhibits spreading, migration, and sprouting of primary brain microvascular endothelial cells. Genetic or immunological inhibition of Nogo-A activity increased density of blood vessels in vivo.	Cultured primary brain-derived microvascular endothelial cells (MVECs) and PC12 cell culture	[37]

Abbreviations: IL-6—Interleukin 6, TNF-α—Tumor necrosis factor α, TRPV1—Transient receptor potential vanilloid 1, LIMK—LIM kinase, D.R.G. culture—Dorsal Root Ganglia Sensory Neuronal culture, Nogo-A KO mice—Nogo-A knock out mice, NgR—Nogo receptor, LINGO-1—leucine rich repeat and Immunoglobin-like domain-containing protein 1, miRNA—microRNA, LTP—long term potentiation, RNAi—RNA interference, anti-Nogo-A Abs—anti-Nogo-A antibodies PC12 cell culture—cell-line from rat pheochromocytoma.

**Table 3:** RTNs and their partners as candidate biomarkers for ND.

Protein	Potential Biomarker	Material	Method	Ref.
RTN4A and NgR1	Pathological hallmarks of MS. Expression of RTN4A and NgR1 reflects demyelinating lesions of MS	Brain tissue	Immunohistochemistry	[70]
RTN4A	Specific diagnostic biomarker for MS.	Human brain tissue CSF	Western blot	[68]
RTN3	Potential biomarker of treatment in MS.	Plasma	Affinity-based proteomic technologies	[71]
LOTUS	Marker of disease activity in MS. Levels of LOTUS, a ligand of Nogo receptor-1 complex, are inversely correlated with disease activity.	Human CSF	Immunoblotting	[72]
RTN4A and RTN4B	Potential biomarker of ALS severity.	Muscle biopsy samples	Western blot	[69]
RTN4A	Expression of RTN4A in muscles may reflect damage of axons and motor neurons in ALS. Genetic ablation of Nogo-A delays disease in ALS mice.	Transgenic mice with G86R murine superoxide dismutase 1 (SOD1) mutation	Western blot, Immunohistochemistry, Immunoprecipitation, RT-PCR	[73]

**Table 4:** The role of RTNs in neurodegenerative diseases.

Protein	Neurodegenerative Disease	Significance in Disease Pathogenesis
RTN4A	Relapsing and progressive MS	Nogo-A present in myelin debris inhibits axonal/myelin repair in MS plaques and leads to degeneration. In acute and chronic MS lesions, Nogo-A limits regeneration and sprouting of damaged axons. Nogo-A, by S1PR2 and the NgR1-LINGO-1-p75/TROY pathway, causes disassembly of the actin and microtubule system with subsequent neuronal growth cone collapse. Inhibits demyelination of oligodendrocytes.
	MS	Modulates immune response in neuroinflamation. Overexpression of Nogo623-640 or Nogo-66 results in the generation of T and B cell response. Strong Th1 response with a release of proinflammatory molecules and antibody targeting of other myelin antigens can lead to inflammation and degeneration.
	MS	Suppresses the action of Nogo-A (e.g., by antibodies anti-NogoA), promotes axonal sprouting, regeneration, and remyelination as well as enhances plasticity.
RTN4A	ALS	Increased immunoreactivity of RTN4 has been found in denervated muscle fibres of patients with ALS. NogoA overexpression destabilises neuromuscular junctions, which may cause nerve terminal retraction and denervation with motor neuron death due to deprivation of tissue neurotrophin resulting in muscle atrophy.
RTN4A, B, C	whole spectrum of disease ALS	In muscle tissue samples from patients with ALS, altered mRNA expression of three forms of RTN4 with a disease-causing dominant mutation has been found. Moreover, increased mRNA of RTN4A and RTN4B was observed in the presymptomatic stage of disease, whereas RTN4C was increased exclusively in asymptomatic animals. RTN4A has been found to be related to a higher risk of ALS development in lower motor neuron syndrome.
RTN4A	ALS	Suppression of RTN4A (e.g., RTN4A deletion) in mice reduces muscle denervation and prolongs survival in a mouse model of ALS.
RTN3	AD	RTN3 reduces BACE1 activity, Aβ generation and amyloid deposition by two pathways. First, augmented interreaction between RTN3 and BACE1 creates spatial blockage that prevents BACE1 from binding to its amyloid-beta precursor protein (APP) substrate for catalytic cleavage. Second, overexpression of RTN3 can arrest BACE1 in ER compartmets, where an almost neutral pH environment is not conducive to Aβ production.
RTN4B/C	AD	Interacts with β-secretase and inhibits production of amyloid beta (Aβ).
RTN4B	AD	Increased expression of RTN4B in hippocampal tissue from aged rat treated with Aβ has been found to be associated with microglial activation and increased caspase 3 activity.
RTN4A	AD	Overexpression of RTN4A promotes Aβ secretion. Nogo-A, by binding to NgR, regulates secretion of amyloid beta-42 through two signalling molecules ROCK and PKC. Activation of ROCK may increase generation of Aβ-42 while induction of PKC may inhibit it.
RTN4	AD	RTN4 as a therapeutic target—inhibition of RTN4 ameliorates learning and memory deficits as well as restores the expression levels of synapto-dendric complexity and axonal sprouting in APP transgenic model of AD in the early/intermediate stage of the disease.

## Data Availability

Not Applicable.
